# Self-assembly at solid surfaces

**DOI:** 10.3762/bjnano.2.91

**Published:** 2011-12-20

**Authors:** Sidney R Cohen, Jacob Sagiv

**Affiliations:** 1 Department of Chemical Research Support, The Weizmann Institute of Science, P.O.B. 26, Rehovot 76100, Israel; 2 Department of Materials and Interfaces, The Weizmann Institute of Science, Rehovot 76100, Israel

The spontaneous formation of highly ordered amphiphilic monolayers on solid surfaces by adsorption from organic solutions at the liquid–solid interface was first reported in the seminal work of W. A. Zisman and co-workers in the mid-20^th^ century [1]. In that work, attention was focused on the remarkable wetting properties of such monolayers, which were not only hydrophobic, but also oleophobic, i.e., they are not wetted by many organic oils, including the solutions from which they were obtained. Several directions of basic study and applications were then pursued employing these *oleophobic monolayers*: Confinement of molecules of interest for surface examination, prevention of spreading of liquids, friction and wear reduction, and surface passivation and protection.

Whereas the early study of such monolayers indeed attracted considerable attention over the years, perhaps their greatest impact was yet to come, in new directions of research that could not have been foreseen at the time. These avenues exploit the ability to finely tune a wide variety of surface properties, for many diverse potential applications, through the combination of molecular self-assembly, chemical design, and postassembly surface manipulation by various chemical and physical techniques. The term *self-assembling monolayer* was thus coined with reference to the planned layer-by-layer assembly of organized films thicker than a single monolayer [2]. These directions, which gained momentum in the 1980s and continue strongly today, are forging new avenues of development. With the advent of relatively recent technologies for small-scale patterning, interest in self-assembled films has seen a surge of activity throughout a wide range of areas ranging from biointerfaces to data storage and devices.

This Thematic Series presents a small, but significant sampling of these exciting areas of research. Thus, the functionality of self-assembled films or of structures derived from them is demonstrated, including mechanical, electrical, and catalytic properties. Unique nanoscale structures are prepared employing lithographic processes and the templating capabilities of the films. Finally, the different characterization techniques employed in these studies point to the unique challenges involved in surface analysis at the nanoscale, and reveal the fascinating properties of the various films and structures.

We hope that this Thematic Series will serve as an inspiration for those wanting to learn about and become involved in the field, and will help expand the horizons of those already engaged in its active research. We would like to thank the Beilstein-Institut for the opportunity to present this Thematic Series, and of course the contributors for their efforts and ingenuity in furthering the research and development of self-assembly at solid surfaces into ever-expanding new areas of scientific activity.

Sidney R. Cohen and Jacob Sagiv

Rehovot, December 2011
